# Efficacy of Acid-Treated Mangosteen Peel as a Broad-Spectrum Mycotoxin Binder

**DOI:** 10.3390/toxins18050215

**Published:** 2026-05-02

**Authors:** Warissara Kasikonsunthonchai, Saowalak Adunphatcharaphon, Chris Elliott, Doris Marko, Dino Grgic, Florian Call, Awanwee Petchkongkaew

**Affiliations:** 1 School of Food Science and Technology, Faculty of Science and Technology, Thammasat University (Rangsit Campus), Pathum Thani 12120, Thailand; mook.coe@tu.ac.th; 2 Center of Excellence in Global Food Security (CoE-GFS), Thammasat University (Rangsit Campus), Pathum Thani 12120, Thailand; chris.elliott@qub.ac.uk; 3 International Joint Research Centre on Food Security (IJC-FOODSEC), 111 Thailand Science Park, Phahonyothin Road, Khong Luang, Pathum Thani 12120, Thailand; s.adunphatcharaphon@hotmail.com; 4 Institute for Global Food Security, School of Biological Sciences, Queen’s University Belfast, Belfast BT9 5DL, Northern Ireland, UK; 5 Department of Food Chemistry and Toxicology, Faculty of Chemistry, University of Vienna, 1010 Vienna, Austria; doris.marko@univie.ac.at (D.M.); dino.grgic@univie.ac.at (D.G.); florian.call@univie.ac.at (F.C.); 6 Doctoral School in Chemistry, University of Vienna, 1010 Vienna, Austria

**Keywords:** agricultural waste, mycotoxin adsorption, mangosteen peel, chemical surface modification, BCG economy

## Abstract

Multiple mycotoxins in feed threaten animal health and food safety, demanding sustainable mitigation strategies. This study evaluated acid-modified mangosteen peel (AMP), an agricultural by-product, as a potential multi-mycotoxin adsorbent. Physicochemical characterization using scanning electron microscopy (SEM), Brunauer–Emmett–Teller (BET) surface area analysis, and Fourier transform infrared spectroscopy (FTIR) analyses demonstrated that acid modification increased surface area (1.9 to 9.03 m^2^/g), pore volume (0.005 to 0.027 cm^3^/g), and surface negativity, indicating enhanced adsorption properties. In vitro binding experiments assessed adsorption of aflatoxin B_1_ (AFB_1_), zearalenone (ZEA), ochratoxin A (OTA), T-2 toxin, deoxynivalenol (DON) and fumonisin B_1_ (FB_1_) under different pH conditions. AMP exhibited high adsorption efficiencies for AFB_1_, ZEA, OTA, and T-2 toxin, particularly at pH 3, whereas DON and FB_1_ showed limited binding. Adsorption behavior was dose-dependent and best described by Langmuir and Freundlich isotherm models. Simulated gastrointestinal digestion indicated stable binding of AFB_1_ and ZEA under gastric conditions, with partial release of some toxins at neutral pH. Cytotoxicity assessment in porcine intestinal epithelial cells (IPEC J2) showed no apparent cytotoxic effects at 0.25–1 mg/mL. Therefore, AMP demonstrated improved multi-mycotoxin adsorption compared to the untreated material and showed no apparent cytotoxic effects in vitro within the tested concentration range, indicating its potential as a promising feed additive candidate.

## 1. Introduction

Mycotoxins are a group of secondary metabolites produced by various fungi that can contaminate a variety of food and feedstuff [1]. Their production is influenced by factors such as moisture, time, temperature, and food or feed substrates [2,3]. Mycotoxin contamination has been frequently detected in agricultural commodities in food chains. To date, several hundred different mycotoxins have been identified globally in grains and other crops [4,5]. Among them, aflatoxins (AFs), deoxynivalenol (DON), ochratoxin A (OTA), zearalenone (ZEA), T-2, and fumonisin B_1_ (FB_1_) are of global concern [6,7], as these toxins can cause both acute and chronic health effects in humans and animals including nephrotoxicity, embryotoxicity, and even carcinogenic effects [6,7,8,9]. The widespread co-contamination of food and feed with multiple mycotoxins is an increasing global issue with serious implications for animal and human health and significant economic consequences [7,10]. According to the World Mycotoxin Survey 2025 [11,12,13], over 80% of feed and raw material samples were contaminated with at least one mycotoxin, while more than 60% exhibited co-contamination with multiple mycotoxins. Among the most prevalent toxins worldwide were FB_1_ (detected in over 80% of samples), DON and ZEA; AFs and OTA were also frequently detected, particularly in samples from Asia and Africa. To mitigate these risks, several countries and organizations worldwide have established limits for mycotoxin levels in food and animal feed. However, these maximum limits typically only account for exposure to individual mycotoxins [7]. For mycotoxin in pig feed, the European Union sets both maximum limits and guidance values to ensure animal health and food safety. According to Directive 2002/32/EC, the maximum permitted concentrations are 0.02 mg/kg for AFB_1_ and 0.05 mg/kg for OTA. Additionally, Commission Recommendation 2006/576/EC provides guidance values of 0.9 mg/kg for DON, 0.1 mg/kg for ZEA, 5 mg/kg for FB_1_, and 0.25 mg/kg for T-2 toxins [14].

To mitigate the adverse effects of mycotoxins, various agents are employed in pig production to reduce mycotoxin exposure. Decontamination strategies aimed at the reduction in mycotoxin contamination are classified into three categories: physical, chemical, and biological. Among these strategies, adsorption is a physical approach that has been successfully used to reduce mycotoxins in feedstuffs. These approaches are regarded as highly practicable due to their high efficiency, operational simplicity, cost-effectiveness, and facile implementation [15]. A wide range of adsorbents, including activated carbon, clay minerals, and synthetic resins, have been commercially applied to bind mycotoxins and thereby mitigate their adverse effects on animal health. In addition, biosorption-based approaches have also been proposed as alternative mitigation strategies aiming to reduce mycotoxins. Vázquez-Durán et al. [16] reported that biosorption is a property of certain biomaterials to bind and concentrate selected ions or molecules from aqueous solutions. However, the limited availability of efficient biomaterials capable of simultaneously adsorbing multiple mycotoxins remains a major challenge. Therefore, the development of new adsorbents for multi-mycotoxin adsorption as feed additives is of great importance for food and feed chain integrity. In a preliminary screening, 25 types of Thai agricultural wastes were evaluated for their capacity to reduce mycotoxin concentrations in a buffer solution at pH 3. Among them, mangosteen peel showed high adsorption capacities for AFB_1_ and OTA with binding efficiencies of more than 90% [17]. Mangosteen (*Garcinia mangostana*), widely consumed in Thailand, is a major commercial fruit with an annual production of approximately 121,168 tons in 2023; however, post-consumption disposal of mangosteen peel generates substantial agro-industrial waste (>2000 tons per year), posing environmental and waste management challenges. Mangosteen peel, a by-product of fruit processing, is rich in polyphenols, tannins, and fiber. These components provide abundant hydroxyl (–OH), carboxyl (–COOH), and aromatic functional groups, which can interact with mycotoxins through hydrogen bonding, π–π interactions, electrostatic attraction, and hydrophobic interactions. These compounds have potential adsorptive capabilities [18,19,20]. The utilization of this agricultural waste as an effective feed additive aligns with the Bio-Circular–Green (BCG) Economy concept, which emphasizes sustainable resource use, waste reduction, and value creation through biotechnological innovation. It is recognized that converting mangosteen peel into a value-added product will help promote the circular economy and support environmentally friendly livestock production. Therefore, the aims of this study are to (i) evaluate the *in vitro* adsorption efficiency of mangosteen peel as a multi-mycotoxin adsorbent, (ii) characterize its physicochemical properties and functional groups related to toxin binding, and (iii) assess its stability and safety *in vitro*.

## 2. Results

### 2.1. Mangosteen Peel Characterization

#### 2.1.1. Scanning Electron Microscopy Coupled with Energy Dispersive X-Ray SPECTROSCOPY (SEM-EDS)

**Figure 1:**
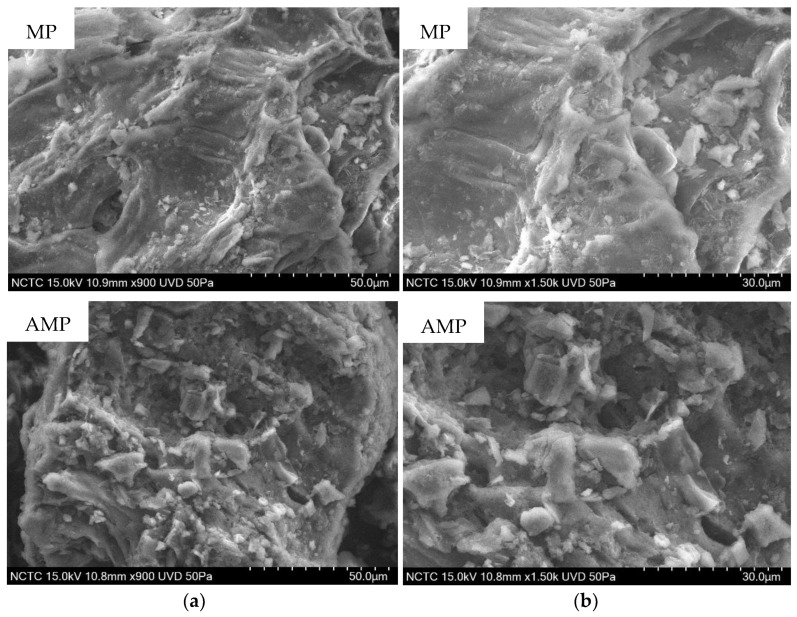
Scanning electron microscopy (SEM) images of mangosteen peel (MP) and acid-treated mangosteen peel (AMP) at magnifications of (**a**) 900× and (**b**) 1500×.

SEM images (Figure 1) revealed significant morphological alterations in MP following sulfuric acid treatment. Untreated MP exhibited a relatively smooth and compact surface, while AMP showed a rougher texture with numerous cavities and porous structures. These surface features likely increased the surface area and created more accessible binding sites for mycotoxins. Moreover, EDS analysis confirmed compositional changes induced by acid treatment, as shown in Table 1. AMP displayed an increase in carbon content and a reduction in oxygen elements.

#### 2.1.2. Fourier Transform Infrared Spectroscopy (FTIR) Characterization

**Figure 2:**
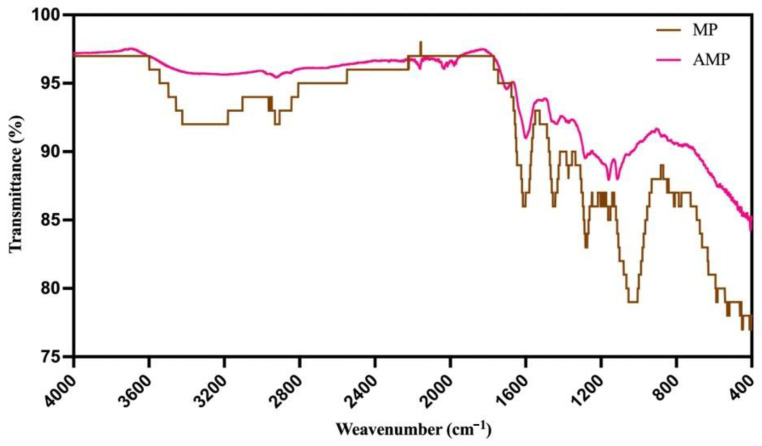
FTIR spectra analysis of MP and AMP.

FTIR analysis of mangosteen peel and sulfuric-treated mangosteen peel was performed in the range of 4000 cm^−1^ to 400 cm^−1^ to analyze the total composition and investigate function groups. FTIR spectra is shown in Figure 2. The FTIR spectrum of MP at 3398 cm^−1^ correspond to stretching vibration of hydrogen bonded (–OH), while bands at 2953 cm^−1^ to 2849 cm^−1^ correspond to asymmetric stretching vibration of hydrocarbon chains in organic compounds (C-H). The band at 1731 cm^−1^ correspond to stretching vibration of the unconjugated carbonyl (C=O) group. Peaks in the 1670–1372 cm^−1^ region are likely associated with C=C stretching vibrations, indicative of the presence of double bonds in the organic structure. The 1273–1155 cm^−1^ range may contain information about C-O and C-C stretching vibrations, possibly related to carbohydrates like cellulose [21,22]. In contrast, the FTIR spectrum of AMP indicates structural modifications due to sulfuric acid treatment with noticeable differences in peak intensity and band shape. A significant reduction in the broad O–H stretching band at 3300 cm^−1^ indicates a decrease in hydroxyl groups, possibly due to the removal of hemicellulose or the protonation effect caused by acid treatment. Peaks at 2978, 2923, and 2847 cm^−1^ correspond to aliphatic C–H stretching, while new or intensified bands at 1979 cm^−1^ and 1648 cm^−1^ suggest increased carbonyl and conjugated double bonds. In addition, the polysaccharide-associated region between 1200 and 1000 cm^−1^ shows a reduction in intensity and slight peak distortion, implying partial disruption of C–O–C and glycosidic linkages of cellulose and hemicellulose.

#### 2.1.3. Zeta Potential

Zeta potential is a measure of the electrostatic charge on the surface of particles, and it influences the interactions between particles and adsorbents. The results indicated that the zeta potential at pH 3 of AMP (−6.82 mV) was more negative than MP (−3.07 mV) at pH 3.

#### 2.1.4. Brunauer–Emmett–Teller (BET) Surface Area Analysis

Brunauer–Emmett–Teller (BET) surface area analysis is a technique used to measure the specific surface area of a material, typically a solid, by determining its adsorption of gas molecules. The surface area, pore size distribution, and total pore volume of MP and AMP were determined by N_2_ adsorption analysis with an ASAP 2460 surface area and porosity analyzer (Micromeritics, USA). The result is shown in Table 2. Based on this result, it appears that AMP (664.55 nm) has a much smaller particle size compared to MP (3156.3). For BET, using a Barrett–Joyner–Halenda (BJH) method, surface area increased from 1.9 m^2^/g to 9.03 m^2^/g. Moreover, the pore volume data underscores the impact of sulfuric acid treatment on porosity. AMP exhibits a significantly higher pore volume (0.027 cm^3^/g) compared to MP (0.005 cm^3^/g), indicating that the treatment has induced a greater number of pores within the material. Although both materials share similar pore diameters (11.2 nm for MP and 11.9 nm for AMP), the substantial increase in pore volume for AMP suggests a more intricate internal structure.

### 2.2. Multiple Mycotoxin Adsorption Efficiency by Modified Mangosteen Peel

**Figure 3:**
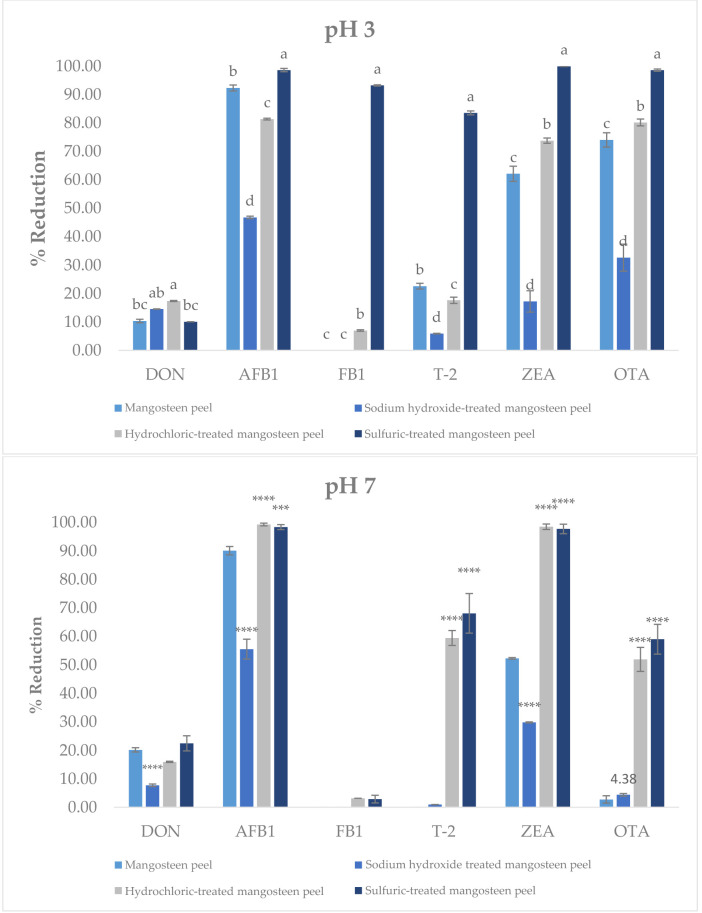
Adsorption efficiency of untreated and chemically modified mangosteen peels toward DON, AFB_1_, FB_1_, T-2, ZEA, and OTA at pH 3 and pH 7. Adsorption was performed using 5 mg adsorbent in 1 mL buffer containing DON and FB_1_ (1 µg/mL) and AFB_1_, T-2, OTA, and ZEA (0.5 µg/mL), incubated at 39.5 °C (250 rpm, 180 min). Residual mycotoxins were quantified by UHPLC–MS/MS. Data are mean ± SD (*n* = 3). Statistical analysis was performed using two-way ANOVA followed by Dunnett’s test (MP as control). Different letters indicate significant differences among adsorbents within each mycotoxin (*p* < 0.05); shared letters (e.g., ab, bc) indicate no significant difference. Asterisks indicate significant differences vs. MP (*** *p* < 0.001, **** *p* < 0.0001).

The adsorption performance of untreated mangosteen peel (MP) and acid-modified mangosteen peel (AMP) was assessed for various regulated mycotoxins. Based on the result shown in Figure 3 at pH 3, MP exhibited considerable affinity toward AFB1 (92.28%), and OTA (74.01%) exhibited moderate and low reduction in ZEA (62.11%) T-2 toxin (22.58%) and DON (10.34%), respectively. However, MP had a lack of reduction for FB_1_. For AMP, the NaOH-treated mangosteen peel demonstrated high reduction in AFB_1_ and OTA of 46.77% and 32.60%, respectively, and showed slight reduction in DON (14.52%), T-2 toxin (5.89%), and ZEA (17.21%) but was ineffective for FB_1_. The HCl-treated mangosteen peel showed improved performance with significant reductions in AFB_1_ (81.34%), ZEA (73.36%), and OTA (80.16%). For DON and T-2, the reduction is similar (17%). Notably, H_2_SO_4_-treated mangosteen peel emerges as the most efficacious, achieving very high reductions across all tested mycotoxins except DON, with reduction rates of 83.52% for T-2, 98.58% for AFB_1_, 99.90% for ZEA, 98.57% for OTA, and 10% for DON. At pH 7, MP showed the capacity to adsorp AFB_1_ and ZEA by more than 50% (90% for AFB_1_ and 52% for ZEA), slight adsorption for DON and OTA (less than 20%), and lack of adsorption in FB_1_ and T-2. However, MP treated with NaOH resulted in the following reduction rates: DON (7.7%), AFB1 (55%), and ZEA (29%). The reduction rate was slightly lower compared to non-treated MP. For AMP, a similar trend was observed, particularly for AFB_1_ and ZEA, where acid treatment achieved nearly complete adsorption for both (more than 97%). For another mycotoxin, H_2_SO_4_-treated MP showed slightly more adsorption than HCl-treated MP. Reduction rates are 22% for DON, 68% for T-2, 58% for OTA, 15% for DON, 59% for T-2, and 51% for OTA in HCl-treated MP. And both acid treatments exhibited FB_1_ reduction of less than 5%. Based on this experiment, the H_2_SO_4_-treated mangosteen peel was selected for future experiments and was referred to as AMP.

### 2.3. pH Effect on Mycotoxin Adsorption

**Figure 4:**
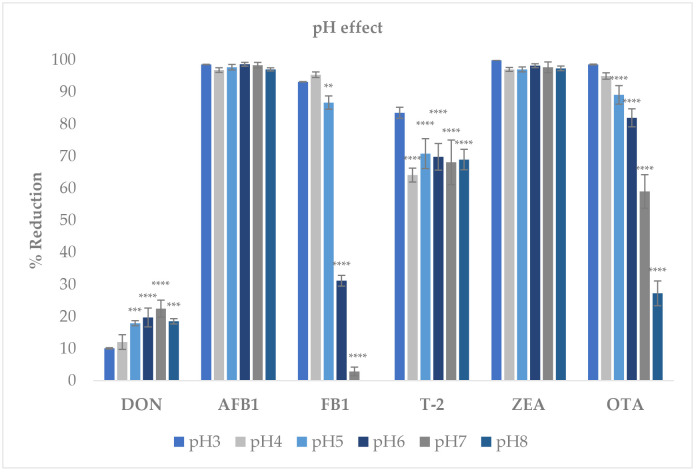
Effect of pH (3–8) on adsorption of DON, AFB_1_, FB_1_, T-2, ZEA, and OTA by acid-treated mangosteen peel (AMP). AMP (5 mg) was incubated with DON and FB_1_ (1 µg/mL) and AFB_1_, T-2, OTA, and ZEA (0.5 µg/mL) in citric–disodium phosphate buffer adjusted to pH 3–8 (39.5 °C, 250 rpm, 180 min). Residual mycotoxins were quantified by UHPLC–MS/MS. Results are expressed as mean ± SD (*n* = 3). Statistical analysis was performed separately for each mycotoxin using one-way ANOVA followed by Dunnett’s multiple comparison test, with pH 3 as the control. Asterisks indicate significant differences compared with pH 3 (** *p* < 0.01, *** *p* < 0.001, **** *p* < 0.0001).

The adsorption efficiency of AMP regarding six regulated mycotoxins (i.e., AFB_1_, ZEA, OTA, FB_1_, T-2 toxin, and DON) was significantly influenced by the pH of the test solution (Figure 4). For DON, the reduction rate increased with increasing pH, ranging from approximately 10% to 19% across pH 3–8. Conversely, as the pH increased, the adsorption efficiency of AMP for the toxins FB_1_, T-2, and OTA decreased markedly, from 93% to 2.87%, 83.52% to 68.94%, and from 98% to 27.22%, respectively. However, AFB_1_ and ZEA appeared to be stable and were not significantly influenced by pH changes under either acidic or basic conditions. The reduction rates for both toxins exceeded 95%.

### 2.4. Effect of Dosage on Mycotoxin Adsorption

**Figure 5:**
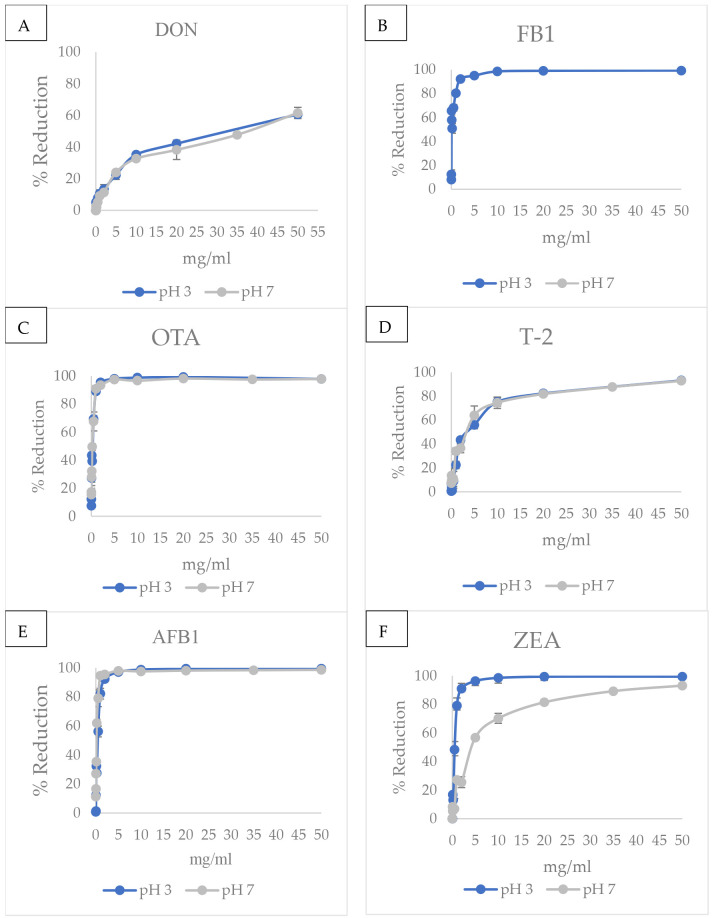
(**A**) Effect of AMP dosage on DON adsorption at pH 3 and pH 7; (**B**) Effect of AMP dosage on FB_1_ adsorption at pH 3 and pH 7; (**C**) Effect of AMP dosage on OTA adsorption at pH 3 and pH 7; (**D**) Effect of AMP dosage on T-2 adsorption at pH 3 and pH 7; (**E**) Effect of AMP dosage on AFB_1_ adsorption at pH 3 and pH 7; (**F**) Effect of AMP dosage on ZEA adsorption at pH 3 and pH 7. AMP (0.01–50 mg/mL) was incubated with DON and FB_1_ (1 µg/mL) and AFB_1_, T-2, OTA, and ZEA (0.5 µg/mL) at 39.5 °C (250 rpm, 180 min). Residual mycotoxins were quantified by UHPLC–MS/MS. Data are mean ± SD (*n* = 3). Dose–response curves were fitted by nonlinear regression and compared between pH 3 and pH 7 using an extra sum-of-squares F test (*p* < 0.05).

As shown in Figure 5, increasing AMP dosage enhanced mycotoxin adsorption, indicating a dose-dependent relationship. For DON, at both pH 3 and 7 levels, reduction increased from low concentrations and reached approximately 70–80% at 50 mg/mL. For FB_1_, AMP achieved rapid and high reduction efficiency, exceeding 90% at concentrations as low as 5–10 mg/mL at pH 3 but a lack of reduction at pH 7. For OTA, a rapid increase in reduction efficiency was observed at low AMP concentrations. Nearly complete reduction was achieved at concentrations above 10 mg/mL, with no noticeable difference between pH 3 and pH 7. For T-2 toxin, AMP showed a sharp increase in reduction efficiency at low concentrations, reaching 80–90% at approximately 10 mg/mL. Both pH conditions demonstrated similar patterns. For AFB_1_, AMP showed near-complete toxin reduction at concentrations as low as 5 mg/mL, with efficiency approaching 100%. For ZEA, AMP reduction capability increased with concentration and reached near-complete reduction at 50 mg/mL. AMP at pH 3 showed slightly higher efficiency than pH 7 at lower concentrations, although the difference diminished at higher concentrations.

### 2.5. Adsorption Isotherm

**Figure 6:**
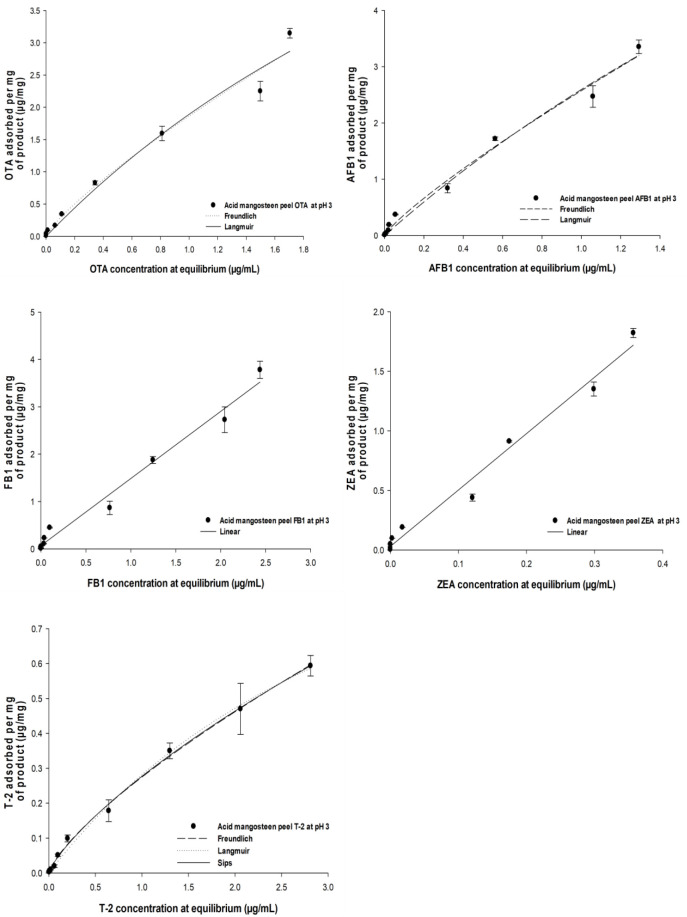
Effect of initial mycotoxin concentration on adsorption efficiency of AMP at pH 3. AMP (0.05%, *w*/*v*) was incubated with increasing concentrations of DON and FB_1_ (0.025–10 µg/mL), AFB_1_ and OTA (0.02–8 µg/mL), and T-2 and ZEA (0.01–4 µg/mL) in citric–disodium phosphate buffer (pH 3) at 39.5 °C (250 rpm). After incubation, residual mycotoxins were quantified by UHPLC–MS/MS. Data are presented as mean ± SD (*n* = 3). Experimental data were fitted to nonlinear Langmuir and Freundlich isotherm models to estimate theoretical maximum adsorption capacity (Ads_max_), distribution coefficients (K_d_), and C_50_ values (see Table 3).

**Figure 7:**
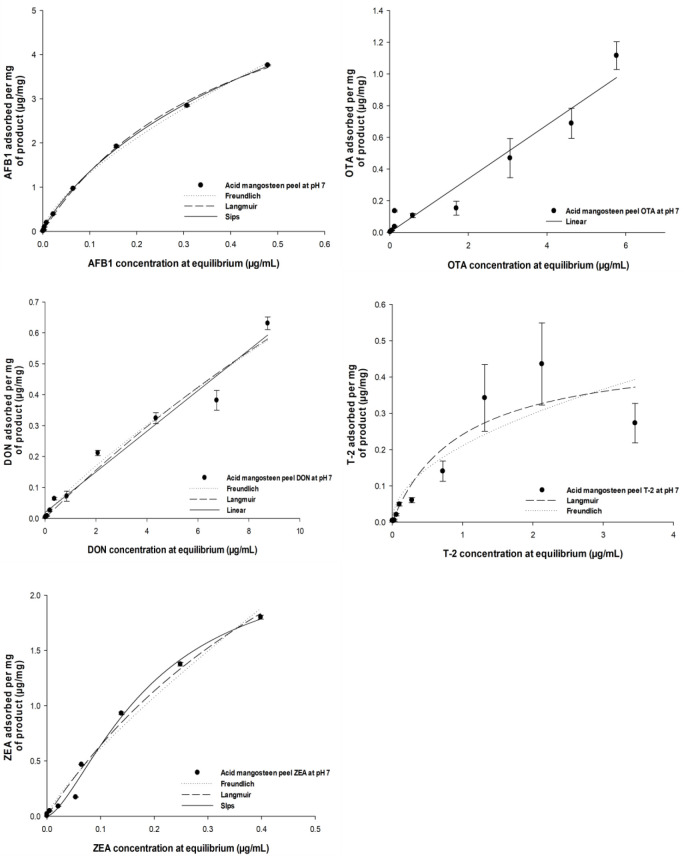
Effect of initial mycotoxin concentration on adsorption efficiency of AMP at pH 7. AMP (0.05%, *w*/*v*) was incubated with increasing concentrations of DON (0.025–10 µg/mL), AFB_1_ and OTA (0.02–8 µg/mL), and T-2 and ZEA (0.01–4 µg/mL) in citric–disodium phosphate buffer (pH 7) at 39.5 °C (250 rpm). After incubation, residual mycotoxins were quantified by UHPLC–MS/MS. Data are presented as mean ± SD (*n* = 3). Nonlinear regression was applied to determine adsorption parameters (Ads_max_, K_d_, and C_50_), summarized in Table 3.

Figure 6 and Figure 7 illustrate adsorption efficiencies across mycotoxin concentrations ranging from 5 to 50 µg/mL. At pH 3 (Figure 6), AMP exhibited higher adsorption efficiencies. For example, AFB_1_ adsorption reached approximately 96.5% at 10 µg/mL and remained above 95% up to 40 µg/mL, indicating strong affinity and site saturation at moderate toxin concentrations. ZEA adsorption exceeded 95% at 15 µg/mL and remained above 90% at 35 µg/mL, while OTA adsorption surpassed 90% at 20 µg/mL. Other mycotoxins such as FB_1_ and T-2 were also adsorbed efficiently, whereas DON exhibited lower adsorption (25% at 40 µg/mL), reflecting weaker interaction with AMP. In contrast, at pH 7 (Figure 7), adsorption efficiencies decreased substantially for most mycotoxins. AFB_1_ adsorption declined to about 75% at 20 µg/mL and further at higher concentrations. ZEA adsorption dropped to below 50% at the same concentration, and OTA and FB_1_ showed moderate adsorption between 60% and 70%. DON adsorption remained consistently low (<20%) throughout the concentration range. The adsorption data were fitted to Langmuir and Freundlich isotherm models, yielding theoretical maximum adsorption capacities (Ads_max_) and distribution coefficients (K_d_), as summarized in Table 3. Ads_max_ values at pH 3 were consistently higher than those at pH 7, indicating enhanced adsorption capacity under acidic conditions. For instance, Ads_max_ for AFB_1_ and ZEA were 103.09% and 104.58%, respectively, suggesting multilayer or cooperative binding likely enabled by increased surface area and functional groups exposed through sulfuric acid treatment. Correspondingly, K_d_ values reflected stronger affinity at pH 3, and AFB1 exhibited a K_d_ of 1.48 µg/mL versus 3.74 µg/mL at pH 7.

### 2.6. Mycotoxin Desorption

To assess the stability of AMP–mycotoxin interactions, desorption experiments were conducted under conditions simulating the gastrointestinal tract, as shown in Table 4. The adsorption experiments were conducted at pH 3, simulating gastric conditions. AMP exhibited a high binding affinity for several mycotoxins: AFB_1_ (95.9%), OTA (97.9%), FB_1_ (91.9%), ZEA (91.4%), and T-2 (57.7%). Following adsorption, desorption was assessed in two phases: a neutral pH buffer (pH 7) to emulate intestinal conditions, and methanol, a strong desorbing solvent, to evaluate the strength and reversibility of the binding interactions.

At pH 7, desorption varied depending on toxin properties. FB_1_ exhibited the highest desorption in buffer at 58.0%, indicating relatively weak retention under physiological pH and suggesting a reliance on electrostatic interactions that diminish as surface charge on AMP decreases. OTA and ZEA showed moderate desorption in buffer (27.2% and 8.5%, respectively), while AFB_1_ remained strongly bound, with only 1.2% released. T-2 desorbed at 16.4% in the first buffer wash, indicating moderate binding stability under neutral conditions. In methanol, desorption levels were generally higher, reflecting disruption of hydrogen bonding and hydrophobic interactions. ZEA exhibited the highest methanol desorption (58.5%), despite its strong initial adsorption, indicating the presence of hydrophobic binding that was susceptible to solvent polarity. OTA and AFB_1_ released 34.3% and 27.6%, respectively, while T-2 showed 26.2% and FB_1_ exhibited minimal MeOH desorption (1.3%), further supporting that FB_1_ binding was pH-sensitive but less affected by organic solvent. Desorption tests indicate the binding stability of biosorbents, crucial for gastrointestinal use. AMP demonstrated minimal desorption (<10%) for AFB_1_ and ZEA under neutral pH conditions.

### 2.7. Assessment of Multi-Mycotoxin Adsorption in Simulated Digestion Model

Table 5 presents the bioaccessibility data for each mycotoxin during the simulated gastric and intestinal digestion phases. AFB_1_ and ZEA exhibited very low bioaccessibility in the gastric phase (7.01% and 6.43%, respectively). However, during the intestinal phase, bioaccessibility slightly increased to 13.18% for AFB_1_ and 56.45% for ZEA, indicating that higher pH conditions facilitated partial desorption of bound toxins. In contrast, DON and FB_1_ showed 100% bioaccessibility in both phases, indicating negligible adsorption by AMP and suggesting that AMP lacked effective interaction mechanisms with these mycotoxins. OTA and T-2 demonstrated intermediate binding, with substantial reductions in bioaccessibility during the gastric phase (66.76% for OTA and 65.65% for T-2), followed by increased levels in the intestinal phase (96.68% and 94.61%, respectively).

### 2.8. Cytotoxicity

**Figure 8:**
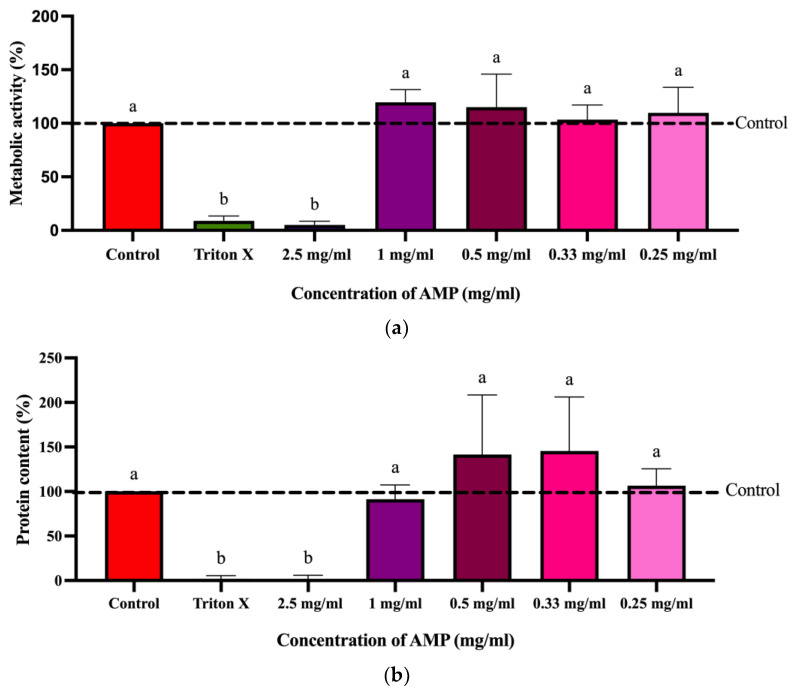
Effects of AMP on cytotoxicity. Impact on the cell viability [%] measured by the CellTiter-Blue (CTB) assay (**a**) and on the cell protein amount [%] measured by the sulforhodamine B (SRB) assay (**b**) of increasing concentrations of AMP after 24 h incubation in IPEC-J2 cells. Values were with reference to the solvent control (water) set to 100%. Results are depicted as mean ± standard deviation of at least five biological replicates (measurements with different cell passages), calculated from the mean value of three technical replicates (repeated measurements with the same cell passage). Outliers after the Nalimov outlier test were excluded. Significant differences in effects between the solvent control and the incubation solutions were calculated by one-sample Student’s *t*-test and significances are indicated with different letters (*p* < 0.05).

The cytotoxic effects of AMP on intestinal porcine epithelial cells from the jejunum of piglets (IPEC-J2) were evaluated using the CellTiter-Blue (CTB) metabolic activity assay and the sulforhodamine B (SRB) total protein assay after 24 h of exposure. As shown in the CTB results (Figure 8), where triton X was used as a positive control to induce cytotoxicity, there was a significant reduction in cell viability (*p* < 0.05) compared with water (solvent control), confirming complete metabolic suppression. AMP at 0.25–1.0 mg/mL showed no significant difference in metabolic activity compared with the solvent control (*p* > 0.05). A reduction in cell viability could only be observed in a concentration of 2.5 mg/mL. As shown in Figure 8b, the SRB assay indicated similar trends in protein content. Triton X significantly reduced total protein content to nearly zero. AMP at 0.25–1.0 mg/mL showed no significant difference compared to the control (*p* > 0.05), and protein levels remained at or above control values (≥100%). However, at a concentration of 2.5 mg/mL, AMP exposure led to a significant reduction in protein content compared to the control group (*p* < 0.05), confirming cytotoxicity at this concentration.

## 3. Discussion

The present study demonstrates that AMP is an effective multi-mycotoxin adsorbent with enhanced adsorption performance compared to MP. The improvement in adsorption capacity can be attributed to pronounced physicochemical modifications induced by acid treatment, which in turn govern toxin–surface interactions. Similar enhancement of adsorption performance following chemical modification has been widely reported for agro-industrial waste-derived biosorbents [16,23,24,25,26]. SEM and BET analyses clearly showed that sulfuric acid treatment transformed MP into a more porous material with a substantially increased surface area from 1.9 to 9.03 m^2^/g and pore volume from 0.005 to 0.027 cm^3^/g. These structural changes are consistent with previous reports demonstrating that acid activation removes amorphous components such as hemicellulose and partially disrupts cellulose structures leading to pore formation and exposure of new binding sites [10,15,27,28]. Comparable increases in surface area and porosity have also been reported for other agricultural waste-derived adsorbents after chemical activation, including fruit peels and lignocellulosic residues [10,16,23,25,28,29,30]. Importantly, the relationship between structure and mycotoxin binding ability can be clearly distinguished between MP and AMP. The untreated MP, characterized by a relatively compact structure, low surface area, and limited pore accessibility, provides fewer active binding sites and restricts toxin diffusion into the internal structure, resulting in lower adsorption efficiency. In contrast, AMP exhibits a more open and porous architecture with increased surface heterogeneity, facilitating both surface adsorption and intra-particle diffusion of mycotoxins. This structural transformation enhances the availability and accessibility of functional groups involved in binding, thereby promoting stronger and more diverse interaction mechanisms. FTIR spectra confirmed the chemical modifications of AMP including reduced hydroxyl band intensity and increased carbonyl-related signals. These changes suggest partial dehydration and formation of conjugated structures, which may favor hydrophobic interactions and hydrogen bonding with non-polar or weakly polar mycotoxins such as AFB_1_ and ZEA. Similar FTIR changes have been described for mangosteen-derived materials and other plant-based biosorbents following chemical treatment [20,21,22,30,31,32]. In addition, the more negative zeta potential of AMP at pH 3 (−6.82 mV) compared with MP (−3.07 mV) indicates enhanced electrostatic attraction toward positively charged or polarizable toxin moieties under acidic conditions. Collectively, these physicochemical alterations provide a mechanistic explanation for the superior adsorption performance of AMP, consistent with observations reported for other agro-waste-based mycotoxin biosorbents [16,32]. AMP exhibited exceptionally high adsorption efficiencies for AFB_1_, ZEA, OTA, and T-2 toxin at pH 3, with reduction rates exceeding 95% for most toxins, while DON remained weakly adsorbed (<20%). This toxin-selective behavior aligns with previous findings that mycotoxin adsorption is strongly influenced by molecular structure, polarity, and ionization state [4,25,33]. In addition to structural factors, adsorption behavior can be further interpreted in terms of the apparent hydrophobicity of mycotoxins under different pH conditions. Under acidic conditions (pH 3), many mycotoxins are predominantly present in a more protonated form, resulting in reduced polarity and increased apparent hydrophobic character. This enhances interactions with hydrophobic domains and aromatic structures on the AMP surface, thereby promoting adsorption. In contrast, at neutral conditions (pH 7), increased molecular polarity and aqueous solubility weaken hydrophobic interactions and may lead to reduced adsorption or partial desorption. Consistent with this interpretation, AFB_1_ and ZEA are relatively hydrophobic molecules, which favor adsorption through hydrophobic interactions and π–π stacking with aromatic structures on carbon-rich surfaces, as reported for several plant-based biosorbents [4,32,33]. OTA, which contains both hydrophobic and ionizable groups, also showed strong binding under acidic conditions, likely due to protonation-enhanced interactions. The pronounced pH effect observed in this study is consistent with earlier reports on biosorbents and clay-based binders [10,25,26]. At low pH, protonation of functional groups on AMP enhances binding affinity, whereas increasing pH weakens electrostatic interactions and promotes partial desorption, particularly for OTA and FB_1_. DON consistently exhibited poor adsorption across all pH levels, which agrees with numerous studies reporting that trichothecenes lack strong functional groups required for effective binding by most adsorbents [26,33,34]. The dose-dependent increase in mycotoxin reduction confirms that AMP adsorption is governed by the availability of active binding sites. Nearly complete adsorption of AFB_1_ and OTA at relatively low AMP dosages (≤10 mg/mL) indicates a high affinity and efficient surface utilization, comparable to that reported for other fruit-derived biosorbents and agricultural by-products [23,32]. Isotherm modeling further supported this observation, with higher Ads_max_ and lower K_d_ values at pH 3 compared with pH 7, indicating stronger and more stable adsorption under acidic conditions. The adsorption behavior of AMP fitted both Langmuir and Freundlich models, indicating the coexistence of monolayer adsorption on homogeneous sites and multilayer adsorption on heterogeneous surfaces. Similar mixed adsorption mechanisms have been reported for modified plant-based adsorbents and activated carbons used for mycotoxin mitigation [16,24,32]. The comparatively low adsorption capacity observed for DON further supports previous reports describing the limited affinity of trichothecenes for most available binders [26,34]. A critical requirement for feed additives is binding stability under gastrointestinal conditions. AMP demonstrated minimal desorption of AFB_1_ and ZEA (<10%) at pH 7, indicating strong and stable binding likely dominated by hydrophobic interactions. In contrast, FB_1_ exhibited substantial desorption in buffer, suggesting that its interaction with AMP is largely electrostatic and, therefore, pH-sensitive. This behavior is consistent with the high polarity and ionic nature of fumonisins, which has been widely reported to limit their stable adsorption by non-clay biosorbents [35,36]. Results from the simulated digestion model corroborated the desorption findings. Low gastric bioaccessibility of AFB_1_ and ZEA confirms effective binding under stomach-like conditions, while increased intestinal bioaccessibility reflects partial release at neutral pH. Such behavior is consistent with previous in vitro digestion studies, which emphasize that the primary protective effect of mycotoxin binders occurs in the upper gastrointestinal tract, thereby limiting intestinal absorption [10,23,32,37,38]. The cytotoxicity assessment using IPEC-J2 cells demonstrated that AMP did not induce apparent cytotoxic effects at concentrations between 0.25 and 1 mg/mL, as evidenced by preserved metabolic activity and protein content. These studies indicate that AMP can be considered non-cytotoxic within this concentration range. In contrast, a reduction in cell viability was observed at 2.5 mg/mL. Similar concentration-dependent responses have been reported for other biosorbents and feed additives, where excessive inclusion levels may induce physical stress or interfere with nutrient availability rather than exerting direct chemical toxicity [26,39,40,41,42]. Accordingly, the decreased viability observed at 2.5 mg/mL is unlikely to reflect intrinsic chemical toxicity of AMP. Instead, excessive solid loading in in vitro systems may increase medium turbidity, promote particle sedimentation onto the cell monolayer, and interfere with nutrient diffusion or serum component availability, thereby affecting metabolic readouts and apparent cell viability [16,25,40,43,44,45]. Such concentration-dependent effects have been described for various bio-adsorbents, where high inclusion levels may influence in vitro outcomes independently of direct cytotoxic mechanisms [45,46]. Furthermore, although phenolic constituents such as xanthones possess antioxidant properties at moderate concentrations [46], higher localized exposure may exert cytostatic or growth-modulating effects in certain cellular models [45,46,47,48]. Taken together, the response observed at 2.5 mg/mL is more plausibly attributed to concentration-dependent physical and microenvironmental effects rather than direct cytotoxic damage. Therefore, AMP demonstrates broader adsorption capacity, particularly for AFB1, ZEA, OTA, and T-2 toxin. While AMP remains less effective for DON and FB1, this limitation is shared by many commercial and experimental binders [4,25,33]. Conventional binders such as aluminosilicates (e.g., bentonite, HSCAS) exhibit high efficacy toward aflatoxins but generally show limited binding capacity for more polar mycotoxins such as DON and FB_1_ [4,25]. Similarly, plant-derived adsorbents, including grape pomace, have demonstrated selective multi-mycotoxin binding, with stronger affinity toward hydrophobic toxins like ZEA and OTA but reduced performance for hydrophilic compounds [33]. Compared with these materials, AMP offers several advantages, including its ability to adsorb multiple structurally diverse mycotoxins, its improved physicochemical properties after acid modification, and its origin from low-cost agricultural waste, supporting sustainable and circular bioeconomy approaches. However, similarly to other biosorbents, AMP still exhibits limitations in binding highly polar mycotoxins such as DON and FB_1_, indicating that no single binder currently provides complete multi-mycotoxin coverage. These studies suggest that AMP could serve as a promising core component in multi-functional or composite binder systems designed to overcome the limitations of individual materials. However, the absence of a direct comparison with a commercial reference binder (e.g., bentonite or activated charcoal) represents a limitation of the present study. Future work should include such benchmarks to enable a more comprehensive evaluation of adsorption performance under comparable conditions.

## 4. Conclusions

AMP demonstrated significantly enhanced multi-mycotoxin adsorption performance compared to untreated mangosteen peel. The acid activation process effectively modified the physicochemical properties of the material, resulting in increased surface area, pore volume, and the exposure of functional groups that favor toxin–surface interactions. Additionally, AMP exhibited strong adsorption capacity across different mycotoxins and maintained stable binding under simulated gastrointestinal conditions, indicating its stability during digestion. Importantly, cytotoxicity assessment using CTB and SRB assays confirmed that AMP is non-toxic to IPEC-J2 intestinal cells at relevant concentrations. Therefore, AMP exhibited no apparent cytotoxic effects under the tested conditions and demonstrates strong potential as an efficient and sustainable mycotoxin adsorbent for application in animal feed. This study also supports the valorization of agricultural waste within a circular bioeconomy framework, contributing to the development of environmentally friendly feed additives. Nevertheless, this study was limited to in vitro evaluations. Therefore, further in vivo studies are required to confirm the efficacy, safety, and practical applicability of AMP under practical animal production conditions.

## 5. Materials and Methods

### 5.1. Materials

One kilogram of mangosteen peel (*Garcinia mangostana*) was obtained from a local farm at Prom Khiri community, Prom Khiri, Nakhon Si Thammarat, Thailand. 

Solid mycotoxin standards (Purity > 98%), including aflatoxin B_1_ (AFB_1_), ochratoxin A (OTA), zearalenone (ZEA), fumonisin B_1_ (FB_1_), T-2 toxin (T-2) and deoxynivalenol (DON) were supplied by Romer Labs, Tulln, Austria. Chemical reagents including sodium chloride (NaCl), potassium chloride (KCl), sodium phosphate (Na_2_HPO_4_), and potassium phosphate monobasic (KH_2_PO_4_) were purchased from Carlo Erba Reagents (Bangkok, Thailand). Methanol (HPLC grade) was purchased from RCI Labscan (Bangkok, Thailand). Other chemical reagents were purchased from Sigma-Aldrich (St. Louis, MO, USA). DI-water was supplied by Milli-Q quality (Millipore, Bedford, MA, USA). For the digestive experiment, all enzymes were supplied by Sigma-Aldrich (Milan, Italy): pepsin from porcine (SRE0001), pancreatin from porcine (P1750), and bile salt.

Cell culture 96-well plates and flasks were obtained from Sarstedt (Nümbrecht, Germany). The cell culture medium, Dulbecco’s Modified Eagle Medium/Nutrient Mixture F-12 (DMEM/F-12; PAN-Biotech, Aidenbach, Germany), along with supplements including fetal bovine serum (FBS), epidermal growth factor (EGF), insulin–transferrin–selenium (ITS), GlutaMAX, and penicillin–streptomycin (P/S) were purchased from Gibco™ (Life Technologies, Carlsbad, CA, USA). Additional reagents, such as sodium bicarbonate (NaHCO_3_) and 4-(2-hydroxyethyl)-1-piperazineethanesulfonic acid (HEPES), were obtained from Sigma-Aldrich (St. Louis, MO, USA). 

### 5.2. Methods

#### 5.2.1. Preparation of Modified Mangosteen Peel (MMP)

##### Mangosteen Peel Preparation

Dried mangosteen peel was washed with water to remove adhering dirt particles, cut into pieces of approximately 1.5 cm × 1.5 cm, then oven dried at 105 °C overnight. The dried samples were ground using a mechanical grinder and sieved through 35-mesh (0.5 mm) and stored in vacuum packaging for further study.

##### Mangosteen Peel Modification

The powder of mangosteen peel was soaked in sulfuric acid (H_2_SO_4_), sodium hydroxy (NaOH), and hydrochloric acid (HCl) to modify mangosteen peel. Then, modified mangosteen peel (MMP) was rested in hood 8 h. They were washed to neutralize with distilled water and dried at 105 °C overnight. The dried samples were ground using a mechanical grinder and sieved through 35-mesh (0.5 mm) and stored in vacuum packaging for further study.

#### 5.2.2. Mycotoxin Standard Preparation

Solid standards of AFB_1_, OTA, ZEA, T-2, and DON was dissolved in methanol (1 mg/mL), while the stock solution of FB_1_ (1 mg/mL) was prepared in methanol/water (50:50, *v*/*v*). These stock solutions were kept in a screw cap vial and stored in the dark at 4 °C. A working solution of multiple mycotoxin standard containing 100 µg/mL of each toxin was prepared by mixing of each stock solution. This was diluted with buffer for use as a working solution in further experiments.

#### 5.2.3. Material Characterization

To understand the morphology, particle size, functional groups, charge, elementary composition, and pore size of the adsorbents, mangosteen peel samples were characterized using scanning electron microscopy coupled with energy dispersive X-ray spectroscopy (SEM-EDS), Fourier transform infrared spectroscopy (FTIR), Brunauer–Emmett–Teller (BET) surface area analysis, and a Zetasizer, respectively.

#### 5.2.4. Assessment of Multiple Mycotoxin Adsorption Efficiency by Modified Mangosteen Peel

Experiments were performed at pH 3 and pH 7 using Citric- Disodium phosphate buffer. Each modified mangosteen peel (5 mg) was suspended in 1 mL of buffer containing multiple mycotoxin standard solution (as discussed in Section 5.2.2). The final concentration of DON and FB_1_ were 1 µg/mL while AFB_1_, T-2, OTA, and ZEA were 0.5 µg/mL. The suspension was thoroughly mixed and further incubated at 39.5 °C with shaking (250 rpm) for 180 min. Following incubation, the sample was centrifuged at 14,000 rpm at 4 °C for 20 min. The supernatant was transferred to an amber glass vial for mycotoxin analysis. Liquid Chromatography–Mass Spectrometer (LC-MS/MS) was used for determination of all residue mycotoxins. Quantification was performed using matrix-matched calibration curves with excellent linearity (R^2^ ≥ 0.998), and method sensitivity was characterized by limits of detection (LOD) and quantification (LOQ) as summarized in Table 6. The percentage mycotoxin reduction was calculated using Equation (1)

(1) $$\mathrm{\% Mycotoxin reduction}=1-\left[\frac{\mathrm{Peak area of mycotoxin sample}}{\mathrm{Peak area of mycotoxin control}}\right]\times 100$$

#### 5.2.5. pH Effect on Mycotoxin Adsorption

From the previous study, H_2_SO_4_-treated mangosteen peel (STMP) was selected as adsorbent. Hereafter, H_2_SO_4_-treated mangosteen peel is referred to as acid-treated mangosteen peel (AMP). The pH effect ranged from 3 to 8. The adsorption capacities of AMP toward AFB_1_, OTA, ZEA, DON, T-2 and FB_1_ were investigated. The working solution of multiple mycotoxin standards containing AFB_1_, OTA, ZEA, DON, T-2 and FB_1_ (DON and FB_1_ were 1 µg/mL whereas AFB_1_, T-2, OTA, and ZEA was 0.5 µg/mL) were prepared in Citric- Disodium phosphate buffer (for pH 3–8). The experiment was performed by adding buffer solution to a vial tube containing 5 mg of AMP. After mixing, the suspensions were incubated at 39.5 °C for 180 min at an agitation speed of 250 rpm. The samples were centrifuged, and the supernatants were analyzed for mycotoxin residuals using UHPLC-MS/MS (Agilent Technologies, Santa Clara, CA, USA).

#### 5.2.6. Material Dosage Effect on Mycotoxin Adsorption

The aim of this experiment is to determine how varying the dosage (amount) of an AMP affects its effectiveness in adsorbing mycotoxins. The material dosage effect can lead to more efficient use of adsorbents in practice, avoiding overuse and ensuring economic viability.

An amount of 0.001–5% dosage of AMP dosages (ranging from 0.01 to 50 mg) was tested for multiple regulated mycotoxin adsorption. The final concentration of DON and FB_1_ were 1 µg/mL whereas another mycotoxin (AFB_1_, T-2, OTA, and ZEA) was 0.5 µg/mL. The experiment was carried out at constant pH (3 and 7) using Citric–Disodium phosphate buffer at a temperature of 39.5 °C with agitation at 250 rpm for 180 min. After that, the samples were centrifuged, and the supernatants were analyzed for mycotoxin residuals using UHPLC-MS/MS (Agilent Technologies, Santa Clara, CA, USA).

#### 5.2.7. Adsorption Isotherm

In this study, the capacity of mycotoxin adsorption was investigated at different initial concentrations of each mycotoxin. Three models were used to predict the amount of mycotoxin adsorbed: the Langmuir, Freundlich, and Sips. Nonlinear regression was used to assess the goodness of fit and further to calculate the parameters involved in the adsorption mechanism (Ads_max_).

Experiments were performed following the method described in Section 5.2.4 The isotherms were tested at a fixed adsorbent dosage with buffer solutions of different mycotoxin concentrations. All tests were performed using a 0.05% dosage (*w*/*v*). The concentrations of mycotoxin were 0.025–10 µg/mL for DON and FB_1_, 0.01–4 µg/mL for T-2 and ZEA, 0.02–8 µg/mL for AFB_1_ and OTA. The AMP was weighed and suspended in 1 mL of each mycotoxin concentration. Following 90 min of incubation, the suspensions were centrifuged at 14,000 rpm for 20 min and supernatant was collected for mycotoxin residual analysis. Data for each mycotoxin adsorption was calculated and fitted to the adsorption isotherm models.

#### 5.2.8. Mycotoxin Desorption

The experiment was tested using 0.5% (*w*/*v*) dosage at a constant temperature of 39 °C with shaking. The AMP (5 mg) was assessed for mycotoxin adsorption at pH 3 by adding 1 mL of multiple mycotoxin standard working solution containing 1 µg/mL of DON and FB_1_ and 0.5 µg/mL of AFB_1_, OTA, T-2 and ZEA. After 90 min of incubation, the suspension was centrifuged, and the supernatant was collected for mycotoxin residual analysis (adsorption). Next, mycotoxin desorption was tested by adding 1 mL of pH 7 phosphate buffer and incubating for 30 min at 39.5 °C with shaking. Afterwards, the suspension was centrifuged, and the supernatant was analyzed for mycotoxin residues. Desorption testing was repeated by adding 1 mL of pH 7 phosphate buffer and incubating for 30 min at 39.5 °C with shaking. Afterwards, the suspension was centrifuged, and the supernatant was analyzed for mycotoxin residues. Desorption testing was also performed in methanol. Methanol containing of multiple mycotoxin solution was used as control and the percentage mycotoxin desorption was calculated using Equation (2).

(2) $$\mathrm{\% Mycotoxin desorption}=\frac{100-(\mathrm{Amount of mycotoxin in solution}\times 100)}{\mathrm{Amount of mycotoxin in control}}$$

#### 5.2.9. Assessment of Multi-Mycotoxin Adsorption in Simulated Digestion Model

The simulated gastrointestinal digestion model was adapted from Adunphatcharaphon et al. [10] and aligned with the standardized INFOGEST [38] static in vitro digestion protocol. The procedure consisted of two sequential phases mimicking the gastric and small intestinal compartments. Simulated gastric fluid (SGF) and simulated intestinal fluid (SIF) were prepared according to the electrolyte compositions described in the INFOGEST method (Table 7).

##### Gastric Phase

An amount of 25 mg of sample was transferred into a reaction tube and mixed with 3.75 mL of SGF. Subsequently, 0.8 mL of pepsin solution (25,000 U/mL), 2.5 μL of 0.3 M CaCl_2_, 100 μL of 1 M HCl (to adjust the pH to 3.0), and 348 μL of distilled water were added to achieve the final electrolyte conditions equivalent to those recommended in the INFOGEST protocol (pH 3). The mixture was vortexed thoroughly and incubated at 39 °C with shaking at 250 rpm for 3 h, simulating gastric digestion.

##### Intestinal Phase

Following gastric digestion, 5 mL of the resulting gastric chyme was transferred into a new tube and combined with 2.75 mL of SIF, 1.25 mL of pancreatin solution (800 U/mL), 0.625 mL of bile salts, 10 μL of 0.3 M CaCl_2_, and 328 μL of distilled water. The pH was adjusted to 7 using 1 M NaOH. The mixture was vortexed and incubated at 39 °C with shaking at 250 rpm for 3 h to complete the intestinal phase.

After finishing the digestive process, the tubes were centrifuged at 10,000 rpm for 20 min. The supernatant was collected and analyzed for residues of each mycotoxin using UHPLC-MS/MS (Agilent Technologies, Santa Clara, CA, USA).

#### 5.2.10. Cytotoxicity Study

##### Cell Line

The intestinal porcine enterocyte cell line (IPEC-J2) was obtained from the Leibniz Institute DSMZ (ACC 701; Braunschweig, Germany). Cells were cultivated in an incubator at 39 °C with 10% CO_2_ for 24 h using a growth medium consisting of DMEM/F12 (1:1), supplemented with 5% fetal bovine serum, 1% insulin–transferrin–selenium, 5 ng/mL epidermal growth factor, 5 mM GlutaMAX, and 16 mM HEPES. Cells between passages 5 and 15 were used for all experiments.

##### AMP Substance Preparing

An amount of 100 mg of AMP was weighed and subjected to a standardized static in vitro gastrointestinal digestion INFOGEST 2.0 protocol [37]. The protocol was performed as described in Call et al. [48] with slight modifications. Briefly, the powder was first reconstituted in 0.5 mL of sterile distilled water to obtain a hydrated matrix suitable for digestion. The mixture was adjusted to a paste-like consistency and subsequently processed through the sequential oral, gastric, and intestinal phases as described by Brodkorb et al. [41]. During the oral phase, AMP was diluted at a 1:1 (*w*/*w*) ratio with simulated salivary fluid (SSF), mixed thoroughly, and incubated at 39 °C for 2 min with salivary amylase. The resulting AMP was then transferred to the gastric phase. For the gastric digestion, an equal volume of SGF was added to achieve a 1:1 (*v*/*v*) dilution. The pH was adjusted to 3, followed by the addition of Rabbit gastric extract (RGE) which was provided by Lipolytech^®^ (Marseille, France) in the final mixture. Samples were incubated at 39 °C for 2 h under continuous mixing. The resulting gastric chyme was subsequently mixed 1:1 (*v*/*v*) with SIF to initiate the intestinal phase, and the pH was adjusted to 7. Bile salts (10 mM final concentration) and pancreatin were added to obtain a trypsin activity of 100 U/mL. Digestion proceeded for 2 h at 39 °C under constant mixing. After finishing the intestinal phase, digests were centrifuged to obtain supernatants. The supernatant fractions were immediately snap-frozen in liquid nitrogen and stored at −80 °C for subsequent use. Prior to cell exposure, thawed AMP digests were diluted in complete IPEC-J2 culture medium to obtain final treatment concentrations corresponding to 1:10, 1:25, 1:50, and 1:100 (*v*/*v*). All dilutions were freshly prepared before experiments to ensure consistency and minimize degradation of digestion-derived constituents.

##### Coupled CellTiter-Blue^®^ and SRB Cytotoxicity Assay

CellTiter-Blue assay was carried out to measure the cell viability, and the protein content was assessed using SRB assay. Both the CellTiter-Blue and SRB assays were conducted with slight modifications based on the method described by Grgic et al. [41]. The assay was conducted in 96-well plates. IPEC-J2 cells (5000 cells per well) were seeded in assay medium and allowed to adhere and grow for 24 h. The medium was then replaced with AMP treatment solutions (as described in section “AMP Substance Preparing”), followed by incubation at 39 °C in a humidified atmosphere containing 10% CO_2_ for an additional 24 h. Following incubation, the supernatants were carefully removed and replaced with 0.1 mL of CellTiter assay medium (1:9, *v*/*v*) in each well containing AMP-treated cells as well as in three cell-free wells serving as blanks. The plates were then incubated in the dark at 39 °C in a humidified atmosphere with 10% CO_2_ for 90 min. Fluorescence was measured using a microplate reader at an excitation wavelength of 560 nm and an emission wavelength of 590 nm (Biotek, Winoosky, VT, USA). For the SRB assay, the CellTiter assay medium was removed from the plates, and 10 µL of cold fixation solution (50% trichloroacetic acid, TCA) was added to each well. The plates were then incubated at 4 °C in the dark for 1 h to allow protein fixation. Subsequently, the fixation solution was discarded, and the wells were washed four times with tap water before being air-dried overnight without lids in the dark. Afterward, the plates were stained with 50 µL SRB-reagent for 60 min without light at room temperature. The staining solution was discarded and washed 2 times with tap water and 2 times with 1% acetic acid solution. The plates were dried at room temperature overnight. The SRB-reagent was solved with 0.1 mL Tris by 5 min orbital shaking in the plate reader and absorbance was measured at 570 nm.

#### 5.2.11. Instrumentation and Chromatographic Conditions

The targeted mycotoxins in each sample were analyzed using an Agilent 1260 infinity II prime LC system equipped with G6400 series LC/MSD triple quadrupole (Agilent Technologies, Santa Clara, CA, USA). The separation was performed using a reverse phase analytical column Gemini^®^ 5 µm C18 110 Å, LC Column 100 × 4.6 mm (Phenomenex, Torrance, CA, USA) at 40 °C. A linear gradient elution was performed using 0.1% formic acid in Milli-Q water (mobile phase A, MPA) and 0.1% formic acid in methanol (mobile phase B, MPB). Both MPA and MPB contain 5 mM ammonium formate. The gradient timetable was set at a flow rate of 1 mL/min, starting with eluting 40% of MPB for 3.0 min, followed by 50% of MPB from 3.10 min to 8.0 min, then an increase to 100% MPB until 9.0 min and a decrease to 40% MPB until 9.1 min. The injection volume was set at 2 μL. The run time and post time were 9.1 and 1.9 min, respectively.

The UHPLC-MS/MS detector was operated in the multiple reaction monitoring (MRM) mode with AJS ESI as the ion source. The QQQ source conditions were as follows: gas temperature, 250 °C; gas flow rate, 8 L/min; nebulizer, 30 psi; capillary voltage, 3300 V; sheath gas temperature and flow set at 350 °C and 12 L/min, respectively, and nozzle voltage was 0 V. Agilent MassHunter Workstation Data Acquisition software version 1.2 build 1.2.23 and Agilent MassHunter Workstation Quantitative Analysis for QQQ version 10.2 build 10.733.8 (Agilent Technology) were used to control the UHPLC-MS/MS system: data acquisition and data processing, respectively. For each mycotoxin, one precursor and at least two product ions were monitored. Selected transition parameters for mycotoxin detection, i.e., precursor ion, product ions, fragmentor, dwell time, collision energy and polarity for each analyte were concluded in Table 8.

#### 5.2.12. Statistical Analysis

All experiments were performed in triplicate and results presented as mean ± standard variation (SD). Statistical program was used for data analysis. Duncan’s Multiple Range Test was used to determine significant differences (*p* < 0.05) among means.

## Figures and Tables

**Table 1:** Elemental analysis of MP before and after sulfuric acid treatment (AMP) (%).

Sample	C	O	S
MP	62.2	35.8	0.1
AMP	72.4	27.1	0.5

**Table 2:** Brunauer–Emmett–Teller (BET), pore characteristics, particle size, and zeta potential of MP and AMP.

Adsorbent	Particle Size (nm)	Pore Volume (cm^3^/g)	Pore Diameter (nm)	Surface Area (m^2^/g)	Zeta Potential (mV)
MP	3156.3	0.005	11.2	1.9	−3.07
AMP	664.55	0.027	11.9	9.03	−6.82

**Table 3:** Theoretical estimates for maximum adsorption (Ads_max_), distribution coefficients (K_d_) and AMP dosage required for 50% mycotoxin reduction (C_50_) for each mycotoxin at pH 3 and pH 7.

Toxin	pH	Ads_max_ (%)	K_d_ (µg/mL)	C_50_ (mg/µg)
AFB_1_	3	103.09	1.48	0.64
	7	100.63	3.74	0.26
ZEA	3	104.58	1.05	0.87
	7	99.04	0.13	8.08
OTA	3	100.79	2.78	0.35
	7	99.59	2.86	0.35
T-2	3	98.63	0.14	7.11
	7	93.88	0.21	5.49
FB_1_	3	100.63	3.74	0.26
	7	nd	nd	-
DON	3	68.26	0.11	25.36
	7	71.39	0.09	27.37

**Table 4:** Mycotoxin desorption under conditions representative of the gastrointestinal tract and strong solvent conditions.

Treatments	Adsorption (%) at pH 3	Desorption (%)		
		1st Buffer pH 7	2nd Buffer pH 7	MeOH
DON	16.9 ± 4.1	4.5 ± 0.8	1.1 ± 0.1	0.8 ± 0.3
AFB_1_	95.9 ± 1.0	1.2 ± 0.7	0.8 ± 0.2	27.6 ± 4.3
FB_1_	91.9 ± 2.2	58.0 ± 12.5	5.6 ± 2.0	1.3 ± 0.2
T-2	57.7 ± 2.9	16.4 ± 1.7	0.9 ± 0.2	26.2 ± 3.6
OTA	97.9 ± 0.2	27.2 ± 0.7	10.0 ± 0.4	34.3 ± 6.1
ZEA	91.4 ± 0.9	8.5 ± 0.9	5.6 ± 1.3	58.5 ± 7.2

**Table 5:** Bioaccessibility of mycotoxin during simulated gastric and intestinal digestion models.

	Bioaccessibility (%)	
	Gastric Phase	Intestinal Phase
DON	100 ± 5.06	100 ± 2.32
AFB_1_	7.01 ± 0.52	13.18 ± 0.3
FB_1_	100 ± 0.59	100 ± 0.54
T-2	65.65 ± 1.01	94.61 ± 0.94
OTA	66.76 ± 0.4	96.68 ± 0.91
ZEA	6.43 ± 0.22	56.45 ± 0.13

**Table 6:** Method validation parameters for multi-mycotoxin analysis by LC–MS/MS.

Mycotoxin	Linearity Range (µg/kg)	Calibration Equation	R^2^	LOD pH 3	LOQ pH 3	LOD pH 7	LOQ pH 7
AFB_1_	0.5–500	y = 1294.17x + 5461.74	0.9982	2.79	9.30	3.88	12.93
DON	0.5–500	y = 8.64x + 61.35	0.9984	11.50	38.33	7.31	24.36
FB1	0.5–500	y = 26.92x − 69.26	0.9998	1.77	5.89	2.49	8.30
T-2	0.5–500	y = 177.81x + 314.01	0.9998	4.82	16.08	8.84	29.48
OTA	0.5–500	y = 168.20x − 295.21	0.9999	2.15	7.17	6.33	21.09
ZEA	0.5–500	y = 45.99x + 227.59	0.9990	1.60	5.32	11.25	37.51

**Table 7:** Preparation of simulated gastric fluid (SGF) and simulated intestinal fluid (SIF) stock solutions. The volumes of each fluid were for a final volume of 500 mL.

Constituents	Stock Conc. (mol/L)	SGF (pH 3)		SIF (pH 7)	
		Vol. of Stock (mL)	Conc. in SSF (mmol/L)	Vol. of Stock (mL)	Conc. in SSF (mmol/L)
KCl	0.5	6.9	6.9	6.8	6.8
KH_2_PO_4_	0.5	0.9	0.9	0.8	0.8
NaHCO_3_	1	12.5	25	42.5	85
NaCl	2	11.8	47.2	9.6	38.4
MgCl_2_(H_2_0)_6_	0.15	0.4	0.1	1.1	0.33
(NH_4_)_2_CO_3_	0.5	0.5	0.5	-	-

**Table 8:** Transitions parameters for mycotoxin detection in MRM mode.

Compound	Precursor Ion (*m*/*z*)	Product Ion (*m*/*z*)	Dwell Time (ms)	Fragmentor (v)	CE (v)	Polarity
AFB_1_	313.3	285.1	30	190	20	(+)
AFB_1_	313.3	241.1	30	190	40	(+)
DON	297.1	249.2	55	120	4	(+)
DON	297.1	231.1	55	80	10	(+)
FB_1_	722.4	352.3	50	200	40	(+)
FB_1_	722.4	334.3	50	200	40	(+)
OTA	404.1	239	30	100	20	(+)
OTA	404.1	221	30	140	36	(+)
T-2	484.2	215.2	30	140	12	(+)
T-2	484.2	185.1	30	140	4	(+)
ZEA	319	301.2	30	130	20	(−)
ZEA	319	275.1	30	130	20	(−)
ZEA	319	205.1	30	130	20	(−)

## Data Availability

The original contributions presented in this study are included in the article. Further inquiries can be directed to the corresponding author.

## Abbreviations

The following abbreviations are used in this manuscript:

AMP	Acid-treated mangosteen peel
SEM	Scanning electron microscopy
BET	Brunauer–Emmett–Teller
FTIR	Fourier transform infrared spectroscopy
AFB1	Aflatoxin B1
ZEA	Zearalenone
OTA	Ochratoxin A
T-2	T-2 toxin
DON	Deoxynivalenol
FB1	Fumonisin B1
EDS	Energy dispersive X-ray spectroscopy
BJH	Barrett–Joyner–Halenda
UHPLC–MS/MS	Ultra-high-performance liquid chromatography–tandem mass spectrometry
MP	Mangosteen peel
PEC-J2	Intestinal porcine epithelial cells from the jejunum of piglets
CTB	CellTiter-Blue assay
SRB	Sulforhodamine B assay
SGF	Simulated gastric fluid
SIF	Simulated intestinal fluid
